# Structural and Extralinguistic Aspects of Code-Switching: Evidence From Papiamentu-Dutch Auditory Sentence Matching

**DOI:** 10.3389/fpsyg.2020.592266

**Published:** 2020-12-22

**Authors:** Luuk Suurmeijer, M. Carmen Parafita Couto, Marianne Gullberg

**Affiliations:** ^1^Leiden University Center for Linguistics, Leiden, Netherlands; ^2^Department of Language Science and Technology, Saarland University, Saarbrücken, Germany; ^3^Leiden Institute for Brain and Cognition, Leiden, Netherlands; ^4^Centre for Languages and Literature, Lund University, Lund, Sweden

**Keywords:** code-switching, Papiamentu, Dutch, switch location, switch directionality, auditory sentence matching

## Abstract

Despite a wealth of studies on effects of switch locations in code-switching (CS), we know relatively little about how structural factors such as switch location and extralinguistic factors such as directionality preferences may jointly modulate CS (cf., Stell and Yapko, 2015). Previous findings in the nominal domain suggest that within-constituent switching (within the noun phrase) may be easier to process than between-constituent switching (a structural effect), and that there may also be directionality effects with switches preferred only in one language direction (an extra-linguistic effect). In this study we examine a different domain, namely how VP-external (preverbal) vs. VP-internal (postverbal) switch location and switch directionality affects the processing of Papiamentu–Dutch mixed subject-verb-object (SVO) sentences. We manipulated switch location (preverbal/postverbal), and directionality of switch (PD/DP) and tested 50 Papiamentu–Dutch bilinguals on an auditory sentence matching task. The results from the mixed conditions showed no effect of switch location. Instead, we found only an effect of directionality and in an unexpected direction for this population, with switches from Dutch to Papiamentu being processed faster than switches from Papiamentu to Dutch regardless of switch location. The results highlight the importance of taking extralinguistic factors into account, but also the challenges of studying CS, particularly in lesser studied speech communities, and the need for a data-driven, cross-disciplinary approach to the study of CS.

## Introduction

Multilinguals often mix their languages within the same conversation or sentence, a phenomenon known as code-switching (CS; cf. Deuchar, 2012). Most researchers agree that CS is rule-governed, like any other expression of an individual's language. As such, much attention has been devoted to unveiling the potential existence of grammatical configurations that may constrain switching within the boundaries of a single sentence or clause (known as intra-sentential or intra-clausal CS, see Myers-Scotton, 1993; Bullock and Toribio, 2009; Backus, 2015; Toribio, 2017; López, 2020, among many others). Indeed, CS is a much-studied phenomenon, both by linguists and psychologists of different theoretical traditions.

Among the issues that emerge in the investigation of intra-sentential CS, two have received special consideration. The first is the need to disentangle supposed universal restrictions on CS from community-specific grammatical structures that may, in turn, be modulated by extra-linguistic factors (Blokzijl et al., 2017; Balam et al., 2020; cf. also Johns et al., 2019). Of comparable importance is to obtain reliable data on bilinguals' production and comprehension of specific switching patterns (cf. Dussias, 2002; Gullberg et al., 2009; Munarriz-Ibarrola et al., 2018; Lipski, 2019). Despite the descriptive richness and ecological validity of naturalistic production data, some researchers argue that corpus data is not exhaustive, that is, it is possible that counterexamples exist that are not attested in the corpus (see Gullberg et al., 2009, for an overview). Acceptability judgments, commonly used in linguistic studies, allow for more control than naturalistic conversational data, but the validity of the technique has also been questioned for CS research, particularly in communities where CS is stigmatized (cf. Parafita Couto et al., 2015; but see Stadthagen-González et al., 2018 for their proposal of combining 2-alternative forced choice tasks and Thurstone's law of comparative judgments). Negative attitudes toward CS can lead bilinguals to reject sentences that they produce or that their linguistic systems would indeed allow (Parafita Couto et al., 2015) or to lower the ratings in the judgment scale (cf., Badiola et al., 2018 for discussion). Thus, in communities where CS is stigmatized, it may be useful to adopt other techniques less sensitive to the pressure of conscious judgments. In particular, implicit techniques that avoid overt metalinguistic judgements and that probe processing may reveal more about the nature of underlying systems (for an overview of such techniques, see Gullberg et al., 2009). The current study uses such an implicit approach.

This study examines how structural and extra-linguistic factors may affect the processing of code-switched SVO sentences in Papiamentu^1^-Dutch bilinguals. The CS literature has long debated the role of structural constraints and switch locations on where a CS can occur. Constraints such as the Government Constraint (Di Sciullo et al., 1986), the Functional Head Constraint (Belazi et al., 1994), and the Constraint on Closed Class Items (Joshi, 1985) all posit that in production switching is preferred between elements which do not hold a government or functional head relation (e.g., between verbs/prepositions and their complements; determiners and the remaining noun phrase, etc.). This predicts that switches are more likely between major constituents (e.g., between subject-NP and VP) than within (e.g., V and object-NP). However, counter-examples, such as switches between determiners and nouns within NPs, have frequently been documented in production (e.g., Parafita Couto and Gullberg, 2017 for an overview and corpus data), and have been shown to be more easily processable in comprehension than switches between major constituents (e.g., Dussias and Courtney, 1994). For example, Dussias and Courtney (1994) used a sentence matching task to investigate the Functional Head Constraint looking at switches between functional heads and their complements in Spanish–English bilinguals. Their results suggested that switches between determiners and the rest of the noun phrase were well-formed, leading to the conclusion that the Functional Head Constraint is too general a restriction. This result was supported by Dussias (1997), who found that switches between heads and complements were read faster than their respective control conditions. Indeed, earlier studies have shown that switches involving nominal constructions where both the determiner and the noun appear in the same language are less frequent than switches where the determiner comes from one language and the noun from the other (Sankoff and Poplack, 1981).

Moreover, directionality effects have also been observed in naturalistic production such that switches between functional and lexical elements generally go in only one direction, from a functional element in language A to a lexical in language B, rather than also from B to A (Blokzijl et al., 2017; Parafita Couto and Gullberg, 2017). It has been suggested that the language of the morpho-syntactic frame or matrix language determines such patterns (Myers-Scotton, 2002). What determines the choice of matrix language is less clear. However, previous research suggests that extralinguistic factors such as language dominance or language status may play a role (Blokzijl et al., 2017; Parafita Couto and Gullberg, 2017). For example, Blokzijl et al. (2017) compared mixed nominal constructions in Spanish–English bilinguals in Miami and in Nicaragua. They found that Spanish determiners were more likely to appear in mixed nominal constructions than English determiners in the Miami data, but the reverse was true in the Nicaragua data. They suggested that the directionality of switches tends to be toward the language with superior social status or the language of power (English in Florida, Spanish in Nicaragua). Hence in both situations, switching went in the direction of the language of prestige.

Such findings underline that CS practices are embedded in the sociocultural and sociohistorical experiences of the bilingual speakers. This gives rise to the question of whether exposure to asymmetries in the directionality of CS in a given community determines how speakers handle switches. Parafita Couto and Stadthagen-González (2017) explored whether speakers' explicit judgements reflected a preference for the asymmetries observed in production, with a focus on determiner-noun switches in Spanish–English bilinguals in the USA, where Spanish determiners tend to occur more frequently than English determiners (Herring et al., 2010; Valdés Kroff, 2016; Blokzijl et al., 2017). Their results indicated that in mixed nominal constructions English determiners were accepted at a similar rate to Spanish determiners, as long as the determiner was from the same language as the matrix language. This suggests that the direction of switching reflected in the asymmetric choice of matrix language in production (here Spanish) does not shape speakers' intuitions. Similar differences are also reported in the Frisian–Dutch community in the Netherlands. Mixing of Dutch (the majority language) into Frisian (the minority language) is common, but mixing of Frisian into Dutch is not (Breuker, 2001). Bosma and Blom (2019) investigated CS frequency and cognitive control in 5- and 6-year-old Frisian–Dutch bilingual children and found that children who code-switched more often from Dutch to Frisian performed better on a cognitive task. However, no such relationship was found in CS from Frisian to Dutch. The directionality effect could not be explained by language dominance. Instead, the authors suggested an effect of usage patterns whereby Frisian–Dutch bilingual speakers speaking Dutch maintain some degree of separation between their two languages, whereas in Frisian they mix the two lexicons and grammars. Community level effects were also reported by Kootstra and Sahin (2018) in their study of the syntactic preferences in dative sentences by Papiamentu–Dutch bilinguals. They reported differences between speakers of Papiamentu in the Netherlands and speakers of Papiamentu in Aruba, leading them to posit that cross-language structural priming can be seen as a link between cross-linguistic interactions in bilingual individuals and contact-induced language change at the community level.

Despite the wealth of studies examining switch locations and directionality separately, we know relatively little about how structural and extralinguistic factors may interact to modulate CS (cf. Stell and Yapko, 2015). And with the emphasis on the nominal domain in much recent work, we specifically know surprisingly little about the processing of CS in VP-external vs. VP-internal positions. This study therefore examines the comprehension of mixed subject-verb-object (SVO) sentences with switches between the subject-NP and the finite verb (VP-external or preverbal switches), and between the finite verb and the object-NP (VP-internal or postverbal switches). We explore the processing of such switches in Papiamentu–Dutch bilinguals to see whether structural processing is modulated by extra-linguistic factors (cf. Johns et al., 2019). We do this using an auditory version of the sentence-matching task (e.g., Freedman and Forster, 1985; Forster and Stevenson, 1987), where results for matching stimuli are analyzed for reaction times, which are usually longer for unacceptable than for acceptable utterances. Section Auditory Sentence Matching Tasks provides a brief overview of this task. In the next section, we present a brief description of Papiamentu–Dutch bilingualism.

### Papiamentu–Dutch Bilingualism

Both Dutch and Papiamentu (an Iberian-lexifier Creole) are spoken on the Caribbean islands Aruba, Bonaire, and Curaçao (the so-called ABC islands). Papiamentu is the first language of more than 80% of the population on the Caribbean islands (Kester, 2011), where it is an official language (alongside Dutch and English) since 2007 (Jacobs and Muysken, 2019). It is also spoken by around 100,000 Antillean migrants who reside in the Netherlands (Jacobs and Muysken, 2019). There are considerable differences between speakers of Papiamentu in the Caribbean islands and in the Netherlands regarding exposure to and use of Dutch. Although Dutch is an official language in the ABC islands, it is argued to only play a minor role in daily communication there (Kook and Narain, 1993; Kouwenberg and Murray, 1994; Vedder and Kook, 2001). This is different for speakers of Papiamentu in the Netherlands, where Dutch plays an important role in daily communication. Kootstra and Sahin (2018) suggest that such differences in the use of Dutch vs. Papiamentu between the ABC islands and the Netherlands may lead to differences in contact-induced change in these communities.

Papiamentu–Dutch CS in the Netherlands has been examined in bilingual parent-child reading interactions, looking both at language choice and functional differentiation between the languages (cf. Muysken et al., 1996; Vedder et al., 1996). Structural aspects of CS between Papiamentu and Dutch in adult interaction in the Netherlands have previously been investigated in conversational production data (Parafita Couto and Gullberg, 2017) and in online comprehension (Pablos et al., 2019). A study of switching patterns between determiners and nouns (Parafita Couto and Gullberg, 2017) drew on a conversational corpus consisting of 3 h of free conversation involving 25 Papiamentu–Dutch bilinguals born in the Caribbean (most in Aruba), but all resident in the Netherlands at the time of recording (Gullberg et al., 2009). The data showed clear directional effects with a preponderance of Papiamentu determiners followed by switches into Dutch nouns, which was interpreted as reflecting Papiamentu dominance. Although all participants reported using both languages to the same extent daily and to habitually CS with other bilinguals, 24 out of the 25 speakers reported that Papiamentu was their “best language.” Papiamentu dominance is also reported in Pablos et al.'s (2019) study. They used event-related brain potentials (ERPs) to measure online comprehension of CS utterances. Even though all their participants reported using both Dutch and Papiamentu on a daily basis, they felt more confident in Papiamentu than in Dutch. It seems that despite differences in the importance of Dutch in everyday life, Papiamentu dominance can still be found in bilingual populations residing in the Netherlands.

### Bilingual Experience, Language Intuitions, and Language Processing

From a grammatical point of view, most CS research to date has involved the search for universal patterns modulated by the influence of language-specific factors (MacSwan, 2009; López, 2020). Until recently, little attention had been paid to the possible role of cultural norms which have become established over the lifetime of the community. However, recent work suggests that switches tend to be toward the language with superior social status in the community (Blokzijl et al., 2017; Parafita Couto and Gullberg, 2017). Psycholinguistically, an exposure-driven account was posited by Valdés Kroff (2016) suggesting that bilingual speakers converge on conventional production patterns in the community. Indeed, Balam et al. (2020) submit, based on intuition data from three Spanish–English bilingual communities, that speakers' intuitions of mixed “do-constructions” are linked to use in their speech communities and do not merely depend on the linguistic properties of the component languages. This is in line with recent work that highlights the important role that language experience plays in bilingual language processing (Beatty-Martínez and Dussias, 2017; Beatty-Martínez et al., 2018). Beatty-Martínez and Dussias (2017) examined how different production choices may predict comprehension difficulty. They report three experiments on two groups of Spanish–English bilinguals who differed in CS experience. Their results indicate that switching costs depend on the type of CS and bilinguals' language experience. Similarly, Adamou and Shen (2019) explored whether there are language switching costs in communities in which CS is frequent, with a specific focus on Romani–Turkish. Their findings indicate that language switching costs in comprehension depend both on the frequency of CS in the community and on exposure to specific lexical items. They take these findings as support for a usage-based approach to bilingual processing, confirming the need to conduct experimental research that takes into account the communicational habits of the participants.

### Auditory Sentence Matching Tasks

Sentence-Matching Tasks (SMT; Forster, 1979; Freedman and Forster, 1985) have been widely used to probe language users' linguistic (mainly grammatical) knowledge and processing. An advantage of these tasks is that they enable the probing of knowledge/processing without asking for explicit metalinguistic judgements about grammaticality or acceptability. SMTs have traditionally been performed in the written modality. Participants are presented with two sentences on a screen, one after the other, and must decide whether they are identical or not. Accuracy and response time (time locked to the presentation of the second sentence in the pair) are usually measured. The underlying assumption is that speakers respond more quickly to identical than to different pairs, and—crucially—faster to grammatical than to ungrammatical pairs. There are various explanations for this difference in response latency to ungrammatical stimuli (e.g., failure to create higher order representations for ungrammatical sentences in Freedman and Forster, 1985; slow down due to the correction of ungrammatical higher order representations in Crain and Fodor, 1987). Whatever the explanation, the empirical findings seem to support the idea that the task reveals something about underlying grammatical representations in both native and non-native language users (e.g., Duffield et al., 2002, 2007; for a critique of STMs in second language studies, see Gass, 2001), and in bilinguals (e.g., Dussias, 1997, 2001; Lipski, 2018).

However, given that CS tends to occur in the spoken rather than the written modality, an auditory task arguably comes closer to the “natural habitat” of CS (cf. Roberts, 2012 for the same argument for non-literate and/or very young participants). The logic is the same as for written SMTs. Participants are presented with two auditory sentences in sequence and must decide whether the pair is identical or not. Response times are longer for decisions on ungrammatical pairs. A key difference between written and auditory SMTs is the risk of potential memory effects. In written SMTs the sentences often stay on the screen, whereas auditory stimuli are transient in nature. It is therefore important to carefully control the duration of auditory stimuli so as not to (over-)tax phonological working memory (cf. Roberts, 2012). Auditory SMTs have been used to study sentence processing in early second language users (Verhagen, 2009) as well as in bilingual sentence processing (Lipski, 2018).

### The current study

The current study set out to examine how structural and extralinguistic factors may modulate the processing of CS in Papiamentu–Dutch bilinguals. Specifically, we examined how a structural factor, switch location (switches in VP-external or preverbal vs. VP-internal or postverbal positions), may interact with an extralinguistic factor, switch directionality (from Papiamentu to Dutch, PD, or from Dutch to Papiamentu, DP) in the processing of Papiamentu–Dutch mixed subject-verb-object (SVO) sentences with switches between the subject-NP and the finite verb, or between the finite verb and the object-NP. We used an auditory sentence matching task to tap into the processing of such structures.

## Methods

### Participants

We recruited 50 self-identifying Papiamentu–Dutch bilinguals between the ages of 14 and 50 (*Mdn*^*age*^ = 22, *SD*^*age*^ = 7; 24 females) residing either in the Netherlands (*n* = 17) or in Curacao (*n* = 33). They were recruited in the social circles of several (under)graduate students enrolled in the BA/MA Linguistics/Latin American Studies at Leiden University, the Netherlands. Participation was voluntary and no remuneration was offered.

Participants were asked to fill in a consent form and a background questionnaire^2^ (see Supplementary Material 1) in either Dutch or Papiamentu. The majority of the participants requested the questionnaire in Dutch (*n* = 31), especially those who had lived in the Netherlands for a long time. The background questionnaire asked participants to roughly estimate the age of acquisition for both languages (before age 4, during primary school, or secondary school), and self-assessed language ability in both languages (from 1 = only a few words to 4 = confident in extended conversation). It also tapped sociolinguistic information such as attitudes to CS and CS habits on a positive-negative Likert scale. Tables 1, 2 summarize demographic background data from 45 participants (other background data missing). Thirty-two participants reported learning Papiamentu before the age of 4, and 28 also learning Dutch before the age of 4, suggesting that early bilingualism characterized the majority of the participants. This state of affairs is reflected in the self-reported ability in both languages with most participants reporting a score of 3 (=fairly confident in extended conversation). A paired samples *t*-test revealed no difference in Dutch and Papiamentu ability in the group [*t*_(44)_ = 0.561, *p* = 0.577]. Moreover, participants on average held a positive view of CS (*M* = 2.2/5) but their self-estimated switching habits yielded a mid score (*M* = 2.9/5), suggesting some variability in switching habits.

**Table 1:** Participants and self-estimated age of acquisition per language.

**Language**	**Before age 4 (*n*)**	**In primary school (age 5–12) (*n*)**	**In secondary school (age 13–18) (*n*)**
Papiamentu	32	8	5
Dutch	28	13	4

**Table 2:** Participants' self-reported measures of language ability in Papiamentu and Dutch, attitudes toward CS, and switching habits.

**Measure**	**Mean**	**SD**
Papiamentu ability (1–4)^*^	3.4	0.8
Dutch ability (1–4)^*^	3.3	0.9
Switching habits (1–5)^∧^	2.9	1.2
Switching attitudes (1–5)^∧^	2.3	1.2

* *Self-assessed scale for ability in both languages: 1 = only some words/expressions, 2 = confident in basic conversation, 3 = fairly confident in extended conversations, 4 = confident in extended conversations*.

∧ *Self-estimate scale 1 = strongly disagree, 2 = disagree, 3 = neutral, 4 = agree, 5 = strongly agree*.

*The question on switching habits was formulated as follows: “In daily conversations, I keep Papiamentu and Dutch separate.” A score of 1 = strongly disagree therefore indicates frequent switching. The question on attitudes to switching was formulated as follows: “People should avoid mixing Dutch and Papiamentu in the same conversation.” A score of 1 = strongly disagree therefore indicates a positive attitude toward switching*.

### Materials

The stimuli for the auditory sentence matching task consisted of SVO sentences (e.g., *The writer writes a letter*) in which switches were introduced between S and V, between V and O, or between S, V, and O. Twenty unique SVO sentences were constructed consisting of five words in sentences with a Dutch verb, and six words in sentences with a Papiamentu verb (cf. Verhagen, 2009 for sentence length in auditory SMTs)^3^. The lexical items in the stimuli could not be selected in standard psycholinguistic ways since corpora do not exist for Papiamentu against which to check for frequency, for example. Instead, lexical selection was guided by the aim to find words where (a) the Papiamentu version was not a cognate or an obvious loan from Dutch; (b) all Dutch words were of common gender to neutralize possible effects stemming from the fact that Dutch has gender but Papiamentu does not. The 20 unique items were rendered in eight different versions matching eight conditions: two monolingual control conditions (monolingual Papiamentu, P, and monolingual Dutch, D, respectively); four experimental conditions with pre- or postverbal switches with switch direction counter-balanced (PD vs. DP); and two additional filler conditions with both pre- and postverbal switches counter-balanced for switch directions (PDP vs. DPD). The permutations resulted in a total of 160 sentences. Eight lists were created containing one version each of the 20 stimulus sentences. Ten filler sentences were added consisting of three monolingual Dutch, three monolingual Papiamentu, and four switched sentence pairs (see Supplementary Material 2 for a complete list). The filler sentence pairs, common to all participants, consisted of sentence pairs with either a language change in the mixed fillers, or a noun change in the monolingual filler items. A further three training items were also constructed. Table 3 exemplifies the materials, and the details and translations of all items can be found in the Supplementary Material 2.

**Table 3:** Test sentences, permutations of the sentence *The writer writes the letter* in Papiamentu (P) and Dutch (D), with the order and language of S, V, and O constituents indicated.

**Condition**	**Stimulus**	**Order**
1	e^P^ escritor^P^ schrijft^D^ de^D^ brief^D^	PDD
2	de^D^ schrijver^D^ schrijft^D^ e^P^ karta^P^	DDP
3	de^D^ schrijver^D^ schrijft^D^ de^D^ brief^D^	DDD
4	e^P^ escritor^P^ schrijft^D^ e^P^ karta^P^	PDP
5	e^P^ escritor^P^ ta^P^ skibi^P^ de^D^ brief^D^	PPD
6	de^D^ schrijver^D^ ta^P^ skibi^P^ e^P^ karta^P^	DPP
7	de^D^ schrijver^D^ ta^P^ skibi^P^ de^D^ brief^D^	DPD
8	e^P^ escritor^P^ ta^P^ skibi^P^ e^P^ karta^P^	PPP

All sentences (experimental, control items, and fillers) were recorded in three sessions by a female native bilingual Papiamentu–Dutch speaker using the stationary recording equipment in the phonetics lab at the Leiden University Center for Linguistics (a Sennheiser MKH416T microphone with Focusrite Scarlett 2i4 (2nd Gen) USB Audio Interface, and the software Adobe, Audition 2.0 CS6). The speaker was instructed to read the sentences at as similar a pace as possible, with neutral stress patterns, and a neutral falling declarative intonation. Using Praat 6.0 (Boersma, 2001) the recordings were edited into individual audio files labeled with a unique ID. The mean duration of each audio file was 2.06 seconds (*SD* = 0.27 s).

The auditory sentence matching task was programmed in PsychoPy 1.81, an open-source Python based software (http://www.psychopy.org/ for more information on the software). Since Papiamentu has several standard orthographies, on-screen instructions were always in Dutch to avoid engaging participants with potentially unfamiliar orthography. The experimental sentence pairs were always identical (correct answer *yes*), whilst two thirds of the filler trials were identical (correct answer *yes*), and one third non-identical (correct answer *no*). Yes/no responses were “1” and “0” on the number row, respectively. Sentence pairs were presented auditorily with a 250 ms interval between sentences. A visual fixation crosshair appeared on the screen during the auditory presentation. Response times were time-locked to the offset of the second sentence. The question, “Are the sentences the same?,” appeared on the monitor to prompt the response. The button press advanced the experiment. A yes/no comprehension question followed each trial to ensure continued focus on the task and to guarantee that participants processed the target stimuli linguistically.

### Procedure

The experiments were conducted both in Leiden, the Netherlands, and in Curaçao, the Antilles, in participants' homes wherever possible or in quiet parts of public libraries. An experimental assistant conducted the experiment. The experiment was administered on a laptop computer with headphones to guarantee optimal sound quality. The participants received written instructions for the experiment on the screen, and were also given oral instructions before the start of the experiment. The language of the oral instruction was mainly Dutch in the Netherlands, and Papiamentu in Curaçao. The experimenter explained that the participants were to listen to 30 pairs of sentences and that their task was to determine whether the two sentences in a pair were the same or not by pushing a button, “1” for yes or “0” for no when prompted.

After participants had provided written consent, they were randomly assigned to one of the eight counterbalanced lists. The experiment always began with three practice trials with explicit feedback to the participant after each response. Questions after the practice trials were answered and clarifications often stressed the need to provide identity judgements rather than correctness judgments. No feedback was given during the experimental trials. The background questionnaire was filled in after completion of the experiment.

Each session lasted ~20 min.

### Predictions

Based on previous findings, we made the following prediction: (1) Preverbal (VP-external) switches are overall more easily processed than postverbal ones regardless of language direction. Drawing on previous findings from the nominal domain (specifically within-NP-switching) showing that Papiamentu–Dutch bilinguals in mixed noun phrases switch mainly from Papiamentu (determiners) to Dutch (nouns; Parafita Couto and Gullberg, 2017), we made the following exploratory prediction: (2) Switches away from Papiamentu into Dutch (PD) will be more easily processed than switches away from Dutch and into Papiamentu (DP) both pre- and post-verbally.

### Data Treatment

First, we excluded the filler items including the two double mixed conditions from analyses. Further, responses to incorrect trials (*n* = 12), trials with response times 3 SDs above the individuals' means (*n* = 21), and responses below 100 ms (*n* = 26) were removed (cf. Baayen and Milin, 2010 for a discussion of data cleaning). This led to the removal of 59 trials (8%), distributed across all remaining conditions (*n* = 10, 19, 4, 14, 5, and 7, respectively), leaving 691 trials for analysis (452 experimental trials, and 239 control trials). Also following Baayen and Milin (2010), we chose to log-normal transform the data before performing statistical analyses. Treatment and analysis of data was performed using the programmes *Python*, version 3.6.5, and *R* version 3.4.4 (R Core Team, 2017).

We analyzed the two monolingual control conditions and the four experimental mixed conditions separately.

## Results

Participants' response accuracy on the sentence matching task was overall at ceiling (691/703 or 98% accurate replies). Tables 4, 5 summarize the mean and median response times in the sentence matching task in the two monolingual control conditions (Table 4) and the four experimental conditions (Table 5), respectively.

**Table 4:** Mean and median response times (in milliseconds) in the monolingual control conditions.

**Language**	**Median RT (ms)**	**Mean RT (ms)**	**SD (ms)**
Dutch	554	647	363
Papiamentu	412	604	507

**Table 5:** Mean and median response times (in milliseconds) in the mixed experimental conditions, P, Papiamentu; D, Dutch.

**Switch location**	**Switch direction**	**Median RT (ms)**	**Mean RT (ms)**	**SD (ms)**
Preverbal	PD	527	642	398
Preverbal	DP	487	608	343
Postverbal	PD	463	721	579
Postverbal	DP	493	572	383

A two-tailed paired samples *t*-test on log-normalized data from the two monolingual conditions revealed no significant difference between the conditions [*t*_(46)_ = −1.46, *p* = 0.15], suggesting that the bilingual participants were equally comfortable in both languages, in line with the not so sensitive measure of their self-reported abilities in the two languages.

We subjected the data from the experimental conditions (452 trials) to mixed-effects regression models in *R* using the lme4 package (Bates et al., 2015) with participant as random effect, and switch location and switch direction as fixed effects. *P*-values for the fixed effects were obtained with likelihood-ratio tests comparing a model with the effect in question to a reduced model. Following this procedure, only the variable directionality had a significant effect on response times [$\chi_{\left(1\right)}^{2}$ = 4.1, *p* = 0.04], such that switching from D to P yielded significantly faster response times than switches from P to D. There was no significant effect for the structural switch location and no interaction between switch location and switch direction (full Tables in Supplementary Material 3).

Although it did not reach significance, we noted a trend in the postverbal switch location such that switches from P to D postverbally yielded the longest RTs (*M* = 721 ms) whereas switches from D to P postverbally yielded the shortest RTs (*M* = 572 ms). This is in contrast to the preverbal switches where directionality seems to have had little effect (*M* = 642 vs. 608 ms).

To examine whether participants' individual characteristics might affect response times, we also ran analyses that included self-reported switching habits, attitudes toward CS, and self-reported ability in Papiamentu and Dutch separately as random effects in models with direction or switch location as fixed effects on RTs^4^. Again, in all cases, the models yielded non-significant results (cf. Supplementary Material 3).

In a *post-hoc* analysis, we also tested whether the geographical place of testing affected response times, given that 33 participants were tested in Curaçao and 17 in the Netherlands. It seemed likely that participants in Curaçao behave differently from participants in the Netherlands (cf. Kootstra and Sahin, 2018; Jacobs and Muysken, 2019). Table 6 summarizes the data in the experimental conditions split by group. However, the analyses showed no significant effect of group on the log-normalized data.

**Table 6:** Mean and median response times (in milliseconds) in the mixed experimental conditions across participant groups tested in Curaçao vs. the Netherlands; P, Papiamentu; D, Dutch.

**Switch location**	**Switch direction**	**Group**	**Median RT (ms)**	**Mean RT (ms)**	**SD (ms)**
Preverbal	PD	Curaçao	554	638	366
		the Netherlands	437	654	491
Preverbal	DP	Curaçao	562	662	353
		the Netherlands	362	462	279
Postverbal	PD	Curaçao	547	752	544
		the Netherlands	362	638	682
Postverbal	DP	Curaçao	520	593	520
		the Netherlands	454	514	228

Finally, to further explore the variation in the data, we examined correlations between response times and self-estimated age of acquisition in the two languages, self-reported ability in the two languages, self-reported switching habits, and attitudes toward code-switching. Table 7 shows the correlation matrix. Response times only correlated positively with attitudes to switching such that the more negative a participant was to CS, the slower the response times in the mixed conditions. No other background variable correlated with RTs. Other correlations that are perhaps not so informative include abilities in both languages correlating negatively with the self-reported age of acquisition of the respective language (i.e., the lower the AoA, the higher the self-reported ability). Ability in Papiamentu and Dutch were also positively correlated.

**Table 7:** Correlation matrix between log-normalized reaction times (logRT) and language ability in Papiamentu and Dutch (Pap_ability/Dutch_ability), estimated age of acquisition in Papiamentu and Dutch (Pap_AoA/Dutch_AoA), attitude toward CS; and CS habits.

		**logRT**	**Pap_ability**	**Dutch_ability**	**Dutch_AoA**	**Pap_AoA**	**Attitude**	**Habit**
logRT	Pearson's *r*	-						
	*p*-value	-						
Pap_ability	Pearson's *r*	0.051	-					
	*p*-value	0.297	-					
Dutch_ability	Pearson's *r*	−0.019	0.178^***^	-				
	*p*-value	0.701	<0.001	-				
Dutch_AoA	Pearson's *r*	0.030	0.027	−0.406^***^	-			
	*p*-value	0.546	0.593	<0.01	-			
Pap_AoA	Pearson's *r*	0.061	−0.420^***^	0.139^**^	−0.156^**^	-		
	*p*-value	0.215	<0.001	0.005	0.002	-		
Attitude	Pearson's *r*	0.104^*^	−0.276^***^	0.036	0.101^*^	0.176^***^	-	
	*p*-value	0.036	<0.001	0.463	0.041	<0.001	-	
Habit	Pearson's *r*	0.058	−0.271^***^	−0.088	0.190^***^	0.346^***^	0.453^***^	-
	*p*-value	0.243	<0.001	0.076	<0.001	<0.001	<0.001	-

* *p < 0.05*,

** p < 0.01, and

*** *p < 0.001*.

## Discussion and Conclusion

This paper examined the potential interaction in processing of CS between a structural constraint on CS, preverbal (VP-external) vs. postverbal (VP-internal) switch location, and an extralinguistic factor, switch directionality (from language A to B vs. from B to A). Using an auditory sentence matching task we tested Papiamentu–Dutch bilinguals on mixed Papiamentu–Dutch CS sentences. The results can be summarized as follows: there was a significant effect of switch directionality such that switches from Dutch to Papiamentu were processed faster than switches from Papiamentu to Dutch. Further, although not a significant interaction, the trend was particularly prominent in postverbal positions. Switch location did not have an independent effect.

The results are surprising in a number of ways. First, there was no independent effect of a structural influence of switch location, but instead only a main effect of switch direction despite the lack of evidence for any language dominance in the population in the self-reported language ability or in the monolingual control conditions. Second, contrary to expectations drawn from previous studies of Papiamentu–Dutch bilinguals in the nominal domain (Parafita Couto and Gullberg, 2017), the directionality effect went in the opposite direction from the predicted, with Dutch to Papiamentu being an easier switch than Papiamentu to Dutch.

First, the lack of a structural effect is surprising, but difficult to comment on since it constitutes a null result. The directionality effect is surprising in view of a lack of dominance in the population. However, the absence of dominance is perhaps not so surprising as it may first seem. Our self-reported measure is clearly not very sensitive. More importantly, given that the bilinguals are dealing with very simple SVO sentences of 5–6 words, the bilingual parsers are not put under great pressure in the monolingual conditions. The experimental conditions, which put the system under some stress, may therefore be more informative regarding possible underlying dominance patterns, explaining why we find a directionality effect. Following Gollan and Ferreira (2009), who showed that processing costs differ under cued vs. voluntary switching conditions such that switch costs are typically greater for the dominant language in cued conditions than in voluntary switching, we might argue that the mixed conditions in our task forces participants to deal with incoming strings with properties they have not chosen themselves. This may explain a directionality effect even in the absence of independent indicators of dominance.

But how do we explain a directionality effect in the opposite direction from the predicted, with heavier processing costs for switches away from Papiamentu to Dutch (especially postverbally) than from Dutch to Papiamentu? Recall that switch patterns in the nominal domain (specifically within-NP switches) show a clear preference in this population for switches from P to D rather than the other way around. One obvious reason for the different outcome here is that the nominal and verbal domains do not behave the same, and that findings from within-constituent switching in the nominal domain do not predict behavior in within- or in between-constituent switching in the verbal domain. Previous research on switching within the nominal domain has not distinguished between cases where the switched nominal construction (NP) occurs in pre- or post-verbal position (Parafita Couto and Gullberg, 2017; Parafita Couto and Stadthagen-González, 2017), whereas this is exactly what is in focus here.

Moreover, if the directionality effect reflects CS patterns in production, then patterns established in communities over time through usage may influence outcomes in studies like these (cf. Valdés Kroff, 2016). It is possible that post-verbal (VP-internal) switching is uncommon in general, and that it is particularly uncommon in this population to switch away from a Papiamentu verb to a Dutch object-NP in production. The results suggest that we should find switches from Dutch verbs to Papiamentu objects to be more frequent (since processed faster) than switches from Papiamentu verbs to Dutch objects (processed more slowly). This is ultimately a matter for a corpus study. The suggestion has support from recent usage-based proposals in the literature on CS. For example, Bosma and Blom (2019), looking at Frisian–Dutch CS, found that the directionality effect observed could not be explained by language dominance alone. Instead, the authors suggested an effect of usage patterns.

Another option to account for the unexpected directionality effect is that production and comprehension do not necessarily align. Although some studies show that production and comprehension data typically do indicate similar patterns (e.g., Beatty-Martínez and Dussias, 2017; Beatty-Martínez et al., 2018), others challenge this view, providing evidence for different patterns across the modalities (Fairchild and Van Hell, 2017). The contradictory evidence for the relationship between production and comprehension suggests that different linguistic domains may behave differently, and that tasks and populations no doubt also affect outcomes.

Our results also revealed that speakers with more negative attitudes toward CS had slower response times in the mixed conditions. This finding adds to the relatively few studies which directly attempt to link attitudes with CS behavior. For example, Redinger (2010) established a statistical link between language attitudes and language behavior in a sociolinguistic investigation of language attitudes and CS in Luxembourg's multilingual education system. Similarly, Parafita Couto et al. (2014) found that acceptability judgments were related to attitudes in their study of Welsh–English adjective-noun order. However, Badiola et al. (2018) examined the effects of CS attitudes in acceptability judgement tasks among Spanish–English bilinguals in the USA, and found that all participants, regardless of attitude, distinguished between all conditions. It has also been shown that although speakers may have a negative attitude toward CS, they may nonetheless produce code-switches (Montes-Alcalá, 2000). Although in general there are doubts about a direct link between self-reported attitudes and actual behavior, attitudes tend to be studied because of the assumption that they can be at the origin of behavior (Bohner, 2001). Further cross-community research is clearly needed on this topic. An anonymous reviewer also points out that the phrasing of the questions about code-switching in the questionnaire (from http://bangortalk.org.uk/; Deuchar et al., 2014) may have reflected a negative attitude toward code-switching as the default. If participants had an “agreement bias” (e.g., Dillman et al., 2014) this may have led to less reported use of CS and less acceptance of CS than if the questions had been phrased differently. This too is something to consider in future studies.

Finally, the data in this study displays substantial individual variation in a heterogeneous population with a somewhat higher rate of loss of trials than normal, and detrimental effects on statistical power as a result. It is possible that the test procedure itself may have contributed to the variability in that it was not optimal for putting participants in a “bilingual mode” (Grosjean, 2001). The Dutch instructions on screen, and the use of Dutch and Papiamentu in the accompanying oral instructions may have accidentally primed one language over the other (e.g., Kootstra et al., 2010; Kootstra and Sahin, 2018). Further to this, the 45 participants varied on a range of dimensions including age, attitudes to CS and CS habits, test location, and were overall not a typical experimental test population. They were not used to participating in experiments (cf. Gollan and Ferreira, 2009). This is clearly not ideal from an experimental viewpoint where homogeneity is at a premium. However, it does highlight the challenges of working with bilingual populations experimentally, and underlines the need for multi-task approaches where the same individuals can provide several kinds of data allowing for within-subject triangulation (cf. Gullberg et al., 2009 for a similar argument). It is especially important if we are to gain insights into more typical bilingual populations than the university students who mostly populate our experimental studies. That said, it will obviously be important and desirable to replicate this study with a more homogeneous population.

In conclusion, the results do not support a simple structural account which assumes that VP-internal switching is always costlier than a VP-external one. Instead, language directionality seems to play a key role to Papiamentu–Dutch bilinguals. Moreover, in the verbal domain the directionality goes in a surprising direction, with a possible structural interaction whereby switch direction may matter more postverbally (VP-internally) than preverbally (VP-externally). The interaction and the directionality effects will both need further exploration. As they stand, the results suggest at the very least that we must consider extralinguistic variables if we are to understand CS. Key to this venture will be a better grasp of distributional usage patterns across different communities in production, and converging evidence from different methodological approaches tapping into both production and comprehension. Bilinguals' experiences clearly matter (e.g., Lipski, 2014; Valdés Kroff, 2016; Beatty-Martínez and Dussias, 2017; Beatty-Martínez et al., 2018; Toribio, 2018; Adamou and Shen, 2019; Balam et al., 2020), but they are not easy to take into account. It is not always clear which aspects of bilinguals' sociolinguistic and cultural experiences matter (and they may differ across communities), and the lack of production corpora add to the methodological challenges in the study of CS. This study has highlighted these challenges. However difficult, we still believe it is necessary that we attempt to tackle them to further our understanding of CS.

## Data Availability Statement

The raw data supporting the conclusions of this article will be made available by the authors, without undue reservation.

## Ethics Statement

For this study written informed consent from all participants (including the teenage participants) was obtained. At the time of testing (2018), consent by the teenage participants' legal guardian was not required. We adhered to the guidelines of Leiden University which at the time did not require parental consent for teenagers, and did not have an ethics committee proper for this kind of research. Such a committee was only installed on September 1, 2019.

## Author Contributions

All authors contributed to the conception and experimental design of the study. LS performed the experiments, collected the data, and helped collate the data together with experimental assistants. LS and MG conducted the analyses. All authors contributed to the interpretation of the results, the writing of the manuscript, and approved the final version of the manuscript for submission.

## Conflict of Interest

The authors declare that the research was conducted in the absence of any commercial or financial relationships that could be construed as a potential conflict of interest.

## Footnotes

^1^The spelling of the language name throughout this paper will be Papiamentu (typical in Curaçao and in many dictionaries and grammars) rather than Papiamento (typical in Aruba) to honor the fact that most of our participants in this study are from Curaçao.

^2^We adapted the questionnaire from the one used at the ESRC Center for Research on Bilingualism at Bangor University to collect the Welsh-English, Spanish–English, and Spanish-Welsh code-switching corpora (cf. http://bangortalk.org.uk/ and Deuchar et al., 2014).

^3^In Papiamentu the present tense-aspect marker is an independent word, *ta;* hence the difference in word count.

^4^Since a few participants had missing data points for these variables (missing data for switching habits *n* = 5 participants, attitudes toward CS *n* = 5, Papiamentu ability *n* = 4, and Dutch ability *n* = 5), models including these variables drew on smaller datasets.
