# Supplementary material for: Structural and Extralinguistic Aspects of Code-Switching: Evidence From Papiamentu-Dutch Auditory Sentence Matching
Source: Front Psychol. 2020 Dec 22;11:592266. doi: 10.3389/fpsyg.2020.592266 (PMC7783355; doi:10.3389/fpsyg.2020.592266)
Supplement: Supplementary file 1 [file Table_1.DOCX]

Supplementary Material

# Supplementary Data – Background questionnaires in Dutch and Papiamentu

**Vragenlijst** Deelnemer nr. .............

*We zouden u erg dankbaar zijn als u ons de volgende achtergrond informatie wilt geven om ons te helpen met ons onderzoek.*

**1.** Bent u: Man ⬜ Vrouw ⬜? **2. Leeftijd**:……………….………

**3.** Wat is op dit moment uw beroep (of als u met pensioen bent of werkloos, wat was het laatste beroep dat u hebt beoefend voordat u met pensioen bent gegaan of werkloos bent geworden)?

............................................................................................................................

**4.** Geef alstublieft aan waar u voor langere perioden hebt gewoond:

**v.b.**: Plaats: *Willemstad, Curaçao* Data: *1982-1993*

Plaats: *Kralendijk, Bonaire* Data: *1993-1999*

Plaats: *Tilburg, Nederland* Data: *1999-2002 +*

Plaats: *Leiden, Nederland* Data: *2002-2005*

Plaats: ………………………………………… Data: ……….…………………

Plaats: ………………………………………… Data: ……….…………………

Plaats: ………………………………………… Data: ……….…………………

Plaats: ………………………………………… Data: ……….…………………

Plaats: ………………………………………… Data: ……….…………………

Plaats: ………………………………………… Data: ……….…………………

**5.** Wat is uw hoogst genoten opleiding?

⬜ Basisonderwijs

⬜ MAVO/VMBO

⬜ MBO

⬜ HAVO

⬜ VWO

⬜ HBO

⬜ Universitair – Bachelor

⬜ Universitair – Master

⬜ Geen

**6.** Vanaf wanneer kunt u Papiamentu spreken?

⬜ Vanaf dat ik 2 jaar of jonger was

⬜ Vanaf dat ik 4 jaar of jonger was

⬜ Vanaf de basisschool

⬜ Vanaf de middelbare school

⬜ Ik heb Papiamentu leren spreken als volwassene

**7.** Vanaf wanneer kunt u Nederlands spreken?

⬜ Vanaf dat ik 2 jaar of jonger was

⬜ Vanaf dat ik 4 jaar of jonger was

⬜ Vanaf de basisschool

⬜ Vanaf de middelbare school

⬜ Ik heb Nederlands leren spreken als volwassene

**8.** Op een schaal van 1 tot 4, hoe goed vind u dat u Papiamentu kunt spreken?

⬜ **1** Ik ken alleen een paar woorden en uitdrukkingen

⬜ **2** Ik kan me met vertrouwen uiten in een basisgesprek

⬜ **3** Ik kan me met wat vertrouwen uiten in uitgebreide gesprekken

⬜ **4** Ik kan me met volle vertrouwen uiten in uitgebreide gesprekken

**9.** Op een schaal van 1 tot 4, hoe goed vind u dat u Nederlands kunt spreken?

⬜ **1** Ik ken alleen een paar woorden en uitdrukkingen

⬜ **2** Ik kan me met vertrouwen uiten in een basisgesprek

⬜ **3** Ik kan me met wat vertrouwen uiten in uitgebreide gesprekken

⬜ **4** Ik kan me met volle vertrouwen uiten in uitgebreide gesprekken

**10.** Welke taal (of talen) heeft uw moeder met u gesproken wanneer u aan het opgroeien was (indien van toepassing)?

⬜ Papiamentu

⬜ Nederlands

⬜ Papiamentu & Nederlands

⬜ Anders (geef a.u.b. aan welke)……………………………

⬜ Niet van toepassing

**11.** Welke taal (of talen) heeft uw vader met u gesproken wanneer u aan het opgroeien was (indien van toepassing)?

⬜ Papiamentu

⬜ Nederlands

⬜ Papiamentu & Nederlands

⬜ Anders (geef a.u.b. aan welke)……………………………

⬜ Niet van toepassing

**12.** Welke taal (of talen) heeft een andere voogd of verzorger met u gesproken wanneer u aan het opgroeien was (indien van toepassing)?

⬜ Papiamentu

⬜ Nederlands

⬜ Papiamentu & Nederlands

⬜ Anders (geef a.u.b. aan welke)……………………………

⬜ Niet van toepassing

**13.** In welke taal (of talen) kreeg u voornamelijk les op de basisschool?

⬜ Papiamentu

⬜ Nederlands

⬜ Papiamentu & Nederlands

⬜ Anders (geef a.u.b. aan welke)……………………………………

**14.** In welke taal (of talen) kreeg u voornamelijk les op de middelbare school?

⬜ Papiamentu

⬜ Nederlands

⬜ Papiamentu & Nederlands

⬜ Anders (geef a.u.b. aan welke)……………………………………

**15.** Maak hieronder een lijst van vijf mensen waarmee u het vaakst mee in uw alledaagse leven spreekt, hetzij persoonlijk of aan de telefoon, bijvoorbeeld uw partner, uw kind, een vriend(in), een collega etc. Noteer daarbij welke talen u het vaakst gebruikt tijdens een gesprek met die persoon, zoals te zien in de voorbeeldtabel.

| **Naam van persoon of relatie** | **Taal meest gesproken met die persoon:**  **(plaats een vinkje in één vakje hieronder voor elke regel)** | | | |
| --- | --- | --- | --- | --- |
|  | **Papiamentu** | **Nederlands** | **Zowel Papiamentu als Nederlands** | **Een andere taal** |
| 1. *Jan* | *✓* |  |  |  |
| 2. *Moeder* |  | *✓* |  |  |
| 3. *Baas* |  |  | *✓* |  |
| 4. *Janneke* |  |  |  | *✓* |
| 5. *Zus* |  | *✓* |  |  |

Vul alstublieft onderstaand tabel in

|  | **Taal meest gesproken met die persoon:**  **(plaats een vinkje in één vakje hieronder voor elke regel)** | | | |
| --- | --- | --- | --- | --- |
|  | **Papiamentu** | **Nederlands** | **Zowel Papiamentu als Nederlands** | **Een andere taal** |
| **1.** |  |  |  |  |
| **2.** |  |  |  |  |
| **3.** |  |  |  |  |
| **4.** |  |  |  |  |
| **5.** |  |  |  |  |

**16.** Hoe zou u Papiamentu als taal op een schaal van 1 tot 5 rangschikken volgens de volgende eigenschappen? Omcirkel één nummer in elke regel.

ouderwets 1 2 3 4 5 modern

onvriendelijk 1 2 3 4 5 vriendelijk

zonder invloed 1 2 3 4 5 invloedrijk
niet inspirerend 1 2 3 4 5 inspirerend

nutteloos 1 2 3 4 5 bruikbaar
lelijk 1 2 3 4 5 mooi

**17.** Hoe zou u Nederlands als taal op een schaal van 1 tot 5 rangschikken volgens de volgende eigenschappen? Omcirkel één nummer in elke regel.

ouderwets 1 2 3 4 5 modern

onvriendelijk 1 2 3 4 5 vriendelijk

zonder invloed 1 2 3 4 5 invloedrijk
niet inspirerend 1 2 3 4 5 inspirerend

nutteloos 1 2 3 4 5 bruikbaar
lelijk 1 2 3 4 5 mooi

**18.** Vind u uzelf voornamelijk**…?**

⬜ Curaçaoënaar

⬜ Bonaireaan

⬜ Arubaan

⬜ Antilliaans

⬜ Nederlandse

⬜ Anders (geef a.u.b. aan wat):……………………………

**19.** In hoeverre bent u het eens met de volgende stelling:

*“In alledaagse gesprekken houd ik de talen Papiamentu en Nederlands gescheiden.”*

⬜ **1** Geheel mee oneens

⬜ **2** Oneens

⬜ **3** Niet eens of oneens

⬜ **4** Eens

⬜ **5** Geheel mee eens

**20.** In hoeverre bent u het eens met de volgende stelling:

*“Mensen moeten het vermijden om Papiamentu en Nederlands met elkaar te mengen in hetzelfde gesprek.”*

⬜ **1** Geheel mee oneens

⬜ **2** Oneens

⬜ **3** Niet eens of oneens

⬜ **4** Eens

⬜ **5** Geheel mee eens

Hartelijk bedankt voor uw tijd en medewerking.

**Kuestionario** Participante no.............

*Nos lo ta hopi buenagradesidu si señor(a) lo por duna nos e siguiente information di señor(a) su pasado pa yuda nos ku nos investigashon.*

**1.** Shon ta: Homber ⬜ Muhé ⬜? **2. Edat**:……………….………

**3.** Kiko ta señor(a) su profeshon na e momentu aki (of si señor(a) ta ku penshon of si señor(a) ta desempleá, kiko tabata e delaster profeshon ku señor(a) tabata tin prome ku señor(a) a baha ku penshon of a bira desempleá)?

............................................................................................................................

**4.** Por fabor indiká na unda señor(a) a biba pa tempu significante:

**v.b.**: Lugá: *Willemstad, Kòrsou* Fecha: *1982-1993*

Lugá: *Kralendijk, Bonèiru* Fecha: *1993-1999*

Lugá: *Tilburg, Hulanda* Fecha: *1999-2002*

Lugá: *Leiden, Hulanda* Fecha: *2002-2005*

Lugá: ………………………………………… Fecha: ……….…………………

Lugá: ………………………………………… Fecha: ……….…………………

Lugá: ………………………………………… Fecha: ……….…………………

Lugá: ………………………………………… Fecha: ……….…………………

Lugá: ………………………………………… Fecha: ……….…………………

Lugá: ………………………………………… Fecha: ……….…………………

**5.** Kua nivel di edukashon ta e nivel supremo ku señor(a) a gosa di dje?

⬜ Enseñansa básiko

⬜ MAVO/VMBO

⬜ MBO

⬜ HAVO

⬜ VWO

⬜ HBO

⬜ Universidat – Bachelor

⬜ Universidat – Master

⬜ Niun

**6.** For di kua tempu señor(a) por papia papiamentu?

⬜ For di mi tabata tin 2 aña of menos

⬜ For di mi tabata tin 4 aña of menos

⬜ For di enseñansa básiko

⬜ For di skol sekundario

⬜ Mi a siña papia papiamentu komo adulto

**7.** For di kua tempu señor(a) por papia hulandes?

⬜ For di mi tabata tin 2 aña of menos

⬜ For di mi tabata tin 4 aña of menos

⬜ For di enseñansa básiko

⬜ For di skol sekundario

⬜ Mi a siña papia hulandes komo adulto

**8.** Kon bon señor(a) ta pensa señor(a) por papia papiamentu riba un eskala di 1 te 4?

⬜ **1** Mi konose un par di palabra ku ekspreshon so

⬜ **2** Mi por ekspresá mi mes ku konfiansa den un kòmbersashon básiko

⬜ **3** Mi por ekspresá mi mes ku un tiki konfiansa den un kòmbersashon amplio

⬜ **4** Mi por ekspresá mi mes ku hopi konfiansa den un kòmbersashon amplio

**9.** Kon bon señor(a) ta pensa señor(a) por papia hulandes riba un eskala di 1 te 4?

⬜ **1** Mi konose un par di palabra ku ekspreshon so

⬜ **2** Mi por ekspresá mi mes ku konfiansa den un kòmbersashon básiko

⬜ **3** Mi por ekspresá mi mes ku un tiki konfiansa den un kòmbersashon amplio

⬜ **4** Mi por ekspresá mi mes ku hopi konfiansa den un kòmbersashon amplio

**10.** Kua lenga(nan) señor(a) su mama tabata papia ku señor(a) ora señor(a) tabata kresiendo (si ta aplikabel)?

⬜ Papiamentu

⬜ Hulandes

⬜ Papiamentu & hulandes

⬜ Otro (por fabor nombra kua)……………………………

⬜ No ta aplikabel

**11.** Kua lenga(nan) señor(a) su tata tabata papia ku señor(a) ora señor(a) tabata kresiendo (si ta aplikabel)?

⬜ Papiamentu

⬜ Hulandes

⬜ Papiamentu & hulandes

⬜ Otro (por fabor nombra kua)……………………………

⬜ No ta aplikabel

**12.** Kua lenga(nan) señor(a) su vogt of kuidadó tabata papia ku señor(a) ora señor(a) tabata kresiendo (si ta aplikabel)?

⬜ Papiamentu

⬜ Hulandes

⬜ Papiamentu & hulandes

⬜ Otro (por fabor nombra kua)……………………………

⬜ No ta aplikabel

**13.** Na kua lenga(nan) señor(a) a haña les durante di señor(a) su enseñansa básiko?

⬜ Papiamentu

⬜ Hulandes

⬜ Papiamentu & hulandes

⬜ Otro (por fabor nombra kua)……………………………

**14.** Na kua lenga(nan) señor(a) a haña les durante di señor(a) su skol sekundario?

⬜ Papiamentu

⬜ Hulandes

⬜ Papiamentu & hulandes

⬜ Otro (por fabor nombra kua)……………………………

**15.** Traha un lista akibou di sinku hende ku señor(a) ta papia ku ne mas tantu den señor(a) su bida di tur dia, sea personalmente of na telefòn, por ehèmpel señor(a) su partner, su yu, un amigu/amiga, un kolega etc. Nota ku esei kua lenga(nan) señor(a) ta usa durante di un kòmbersashon ku e persona ei, manera den e tabèl di ehèmpel.

| **Nomber di persona of relashon** | **Lengá mas papiá ku e persona ei:**  **(marka e den e vak pa tur persona of relashon)** | | | |
| --- | --- | --- | --- | --- |
|  | **Papiamentu** | **Hulandes** | **Tantu papiamentu komo hulandes** | **Un otro lenga** |
| 1. *Jan* | *✓* |  |  |  |
| 2. *Moeder* |  | *✓* |  |  |
| 3. *Baas* |  |  | *✓* |  |
| 4. *Janneke* |  |  |  | *✓* |
| 5. *Zus* |  | *✓* |  |  |

Por fabor yena e tabèl akibou

| **Nomber di persona of relashon (usa nomber fiktisio si ta nesesario)** | **Lenga mas papiá ku e persona ei:**  **(marka e den e vak pa tur persona of relashon)** | | | |
| --- | --- | --- | --- | --- |
|  | **Papiamentu** | **Hulandes** | **Tantu papiamentu komo hulandes** | **Un otro lenga** |
| **1.** |  |  |  |  |
| **2.** |  |  |  |  |
| **3.** |  |  |  |  |
| **4.** |  |  |  |  |
| **5.** |  |  |  |  |

**16.** Kon lo señor(a) pone papiamentu komo lenga riba un eskala di 1 te 5 sigun e siguiente karakterístikanan? Sirkulá un number riba tur liña.

antikuá 1 2 3 4 5 modèrnu

desagradabel 1 2 3 4 5 agradabel

sin influensha 1 2 3 4 5 influyente
sin inspirashon1 2 3 4 5 inspirá

inútil 1 2 3 4 5 utilisabel
mahos 1 2 3 4 5 bunita

**17.** Kon lo señor(a) pone hulandes komo lenga riba un eskala di 1 te 5 sigun e siguiente karakterístikanan? Sirkulá un number riba tur liña.

antikuá 1 2 3 4 5 modèrnu

desagradabel 1 2 3 4 5 agradabel

sin influensha 1 2 3 4 5 influyente
sin inspirashon1 2 3 4 5 inspirá

inútil 1 2 3 4 5 utilisabel
mahos 1 2 3 4 5 bunita

**18.** Kon señor(a) ta sinti su mes prinsipalmente?

⬜ Kurasoleño

⬜ Bonerianu

⬜ Rubiano

⬜ Antiano

⬜ Hulandes

⬜ Otro (por fabor nombra kua)……………………………

**19.** Den ki medida señor(a) ta di akuerdo ku e siguiente:

*“Den kòmbersashon di tur dia mi ta tene e lenganan papiamentu i hulandes separá.”*

⬜ **1** Mi no ta kompletamente di akuerdo

⬜ **2** Mi no ta di akuerdo

⬜ **3** Mi ta neutral

⬜ **4** Mi ta di akuerdo

⬜ **5** Mi ta kompletamente di akuerdo

**20.** Den ki medida señor(a) ta di akuerdo ku e siguiente:

*“Hende mester evitá di usa papiamentu i hulandes den un kòmbersashon.”*

⬜ **1** Mi no ta kompletamente di akuerdo

⬜ **2** Mi no ta di akuerdo

⬜ **3** Mi ta neutral

⬜ **4** Mi ta di akuerdo

⬜ **5** Mi ta kompletamente di akuerdo

Masha danki pa señor(a) su tempu i koperashon.

# Supplementary Figures and Tables

For more information on Supplementary Material and for details on the different file types accepted, please see [here](http://home.frontiersin.org/about/author-guidelines#SupplementaryMaterial). Figures, tables, and images will be published under a Creative Commons CC-BY licence and permission must be obtained for use of copyrighted material from other sources (including re-published/adapted/modified/partial figures and images from the internet). It is the responsibility of the authors to acquire the licenses, to follow any citation instructions requested by third-party rights holders, and cover any supplementary charges.
