# Supplementary material for: Structural and Extralinguistic Aspects of Code-Switching: Evidence From Papiamentu-Dutch Auditory Sentence Matching
Source: Front Psychol. 2020 Dec 22;11:592266. doi: 10.3389/fpsyg.2020.592266 (PMC7783355; doi:10.3389/fpsyg.2020.592266)
Supplement: Supplementary file 2 [file Table_2.PDF]

F = Filler trial
P = Practice trial
# = Target trial

ID	set	stimulus1	stimulus2	subject	verb	object	translation
1	1	e escritor schrijft de brief	e escritor schrijft de brief	p	d	d	The writer writes the letter
2	1	de schrijver schrijft e karta	de schrijver schrijft e karta	d	d	p	The writer writes the letter
3	1	de schrijver schrijft de brief	de schrijver schrijft de brief	d	d	d	The writer writes the letter
4	1	e escritor schrijft e karta	e escritor schrijft e karta	p	d	p	The writer writes the letter
5	1	e escritor ta skibi de brief	e escritor ta skibi de brief	p	p	d	The writer writes the letter
6	1	de schrijver ta skibi e karta	de schrijver ta skibi e karta	d	p	p	The writer writes the letter
**7**	1	de schrijver ta skibi de brief	de schrijver ta skibi de brief	d	p	d	The writer writes the letter
8	1	e escritor ta skibi e karta	e escritor ta skibi e karta	p	p	p	The writer writes the letter
9	2	e alumno snijdt de ui	e alumno snijdt de ui	p	d	d	The pupil cuts the onion
10	2	de leerling snijdt e siboyo	de leerling snijdt e siboyo	d	d	p	The pupil cuts the onion
11	2	de leerling snijdt de ui	de leerling snijdt de ui	d	d	d	The pupil cuts the onion
12	2	e alumno snijdt e siboyo	e alumno snijdt e siboyo	p	d	p	The pupil cuts the onion
13	2	e alumno ta kòrta de ui	e alumno ta kòrta de ui	p	p	d	The pupil cuts the onion
14	2	de leerling ta kòrta e siboyo	de leerling ta kòrta e siboyo	d	p	p	The pupil cuts the onion
15	2	de leerling ta kòrta de ui	de leerling ta kòrta de ui	d	p	d	The pupil cuts the onion
16	2	e alumno ta kòrta e siboyo	e alumno ta kòrta e siboyo	p	p	p	The pupil cuts the onion
17	3	e hòmber vouwt de trui	e hòmber vouwt de trui	p	d	d	The man folds the sweater
18	3	de man vouwt e suèter	de man vouwt e suèter	d	d	p	The man folds the sweater
19	3	de man vouwt de trui	de man vouwt de trui	d	d	d	The man folds the sweater
20	3	e hòmber vouwt e suèter	e hòmber vouwt e suèter	p	d	p	The man folds the sweater
21	3	e hòmber ta dobla de trui	e hòmber ta dobla de trui	p	p	d	The man folds the sweater
22	3	de man ta dobla e suèter	de man ta dobla e suèter	d	p	p	The man folds the sweater
23	3	de man ta dobla de trui	de man ta dobla de trui	d	p	d	The man folds the sweater
24	3	e hòmber ta dobla e suèter	e hòmber ta dobla e suèter	p	p	p	The man folds the sweater
25	4	e tata roept de buurman	e tata roept de buurman	p	d	d	The father calls the neighbour
26	4	de vader roept e bisiña	de vader roept e bisiña	d	d	p	The father calls the neighbour
27	4	de vader roept de buurman	de vader roept de buurman	d	d	d	The father calls the neighbour
28	4	e tata roept e bisiña	e tata roept e bisiña	p	d	p	The father calls the neighbour
29	4	e tata ta yama de buurman	e tata ta yama de buurman	p	p	d	The father calls the neighbour
30	4	de vader ta yama e bisiña	de vader ta yama e bisiña	d	p	p	The father calls the neighbour

31	4	de vader ta yama de buurman	de vader ta yama de buurman	d	p	d	The father calls the neighbour
32	4	e tata ta yama e bisiña	e tata ta yama e bisiña	p	p	p	The father calls the neighbour
33	5	e ladron steelt de vis	e ladron steelt de vis	p	d	d	The thief steals the fish
34	5	de dief steelt e piská	de dief steelt e piská	d	d	p	The thief steals the fish
35	5	de dief steelt de vis	de dief steelt de vis	d	d	d	The thief steals the fish
36	5	e ladron steelt e piská	e ladron steelt e piská	p	d	p	The thief steals the fish
37	5	e ladron ta hòrta de vis	e ladron ta hòrta de vis	p	p	d	The thief steals the fish
38	5	de dief ta hòrta e piská	de dief ta hòrta e piská	d	p	p	The thief steals the fish
39	5	de dief ta hòrta de vis	de dief ta hòrta de vis	d	p	d	The thief steals the fish
40	5	e ladron ta hòrta e piská	e ladron ta hòrta e piská	p	p	p	The thief steals the fish
41	6	e kunukero drinkt de melk	e kunukero drinkt de melk	p	d	d	The farmer drinks the milk
42	6	de landbouwer drinkt e lechi	de landbouwer drinkt e lechi	d	d	p	The farmer drinks the milk
43	6	de landbouwer drinkt de melk	de landbouwer drinkt de melk	d	d	d	The farmer drinks the milk
44	6	e kunukero drinkt e lechi	e kunukero drinkt e lechi	p	d	p	The farmer drinks the milk
45	6	e kunukero ta bebe de melk	e kunukero ta bebe de melk	p	p	d	The farmer drinks the milk
46	6	de landbouwer ta bebe e lechi	de landbouwer ta bebe e lechi	d	p	p	The farmer drinks the milk
47	6	de landbouwer ta bebe de melk	de landbouwer ta bebe de melk	d	p	d	The farmer drinks the milk
48	6	e kunukero ta bebe e lechi	e kunukero ta bebe e lechi	p	p	p	The farmer drinks the milk
49	7	e kachó bijt de schoen	e kachó bijt de schoen	p	d	d	The dog bites the shoe
50	7	de hond bijt e sapatu	de hond bijt e sapatu	d	d	p	The dog bites the shoe
51	7	de hond bijt de schoen	de hond bijt de schoen	d	d	d	The dog bites the shoe
52	7	e kachó bijt e sapatu	e kachó bijt e sapatu	p	d	p	The dog bites the shoe
53	7	e kachó ta mòrde de schoen	e kachó ta mòrde de schoen	p	p	d	The dog bites the shoe
54	7	de hond ta mòrde e sapatu	de hond ta mòrde e sapatu	d	p	p	The dog bites the shoe
55	7	de hond ta mòrde de schoen	de hond ta mòrde de schoen	d	p	d	The dog bites the shoe
56	7	e kachó ta mòrde e sapatu	e kachó ta mòrde e sapatu	p	p	p	The dog bites the shoe
57	8	e kasadó schiet de pijl	e kasadó schiet de pijl	p	d	d	The hunter shoots the arrow
58	8	de jager schiet e flecha	de jager schiet e flecha	d	d	p	The hunter shoots the arrow
59	8	de jager schiet de pijl	de jager schiet de pijl	d	d	d	The hunter shoots the arrow
60	8	e kasadó schiet e flecha	e kasadó schiet e flecha	p	d	p	The hunter shoots the arrow
61	8	e kasadó ta tira de pijl	e kasadó ta tira de pijl	p	p	d	The hunter shoots the arrow
62	8	de jager ta tira e flecha	de jager ta tira e flecha	d	p	p	The hunter shoots the arrow
63	8	de jager ta tira de pijl	de jager ta tira de pijl	d	p	d	The hunter shoots the arrow
64	8	e kasadó ta tira e flecha	e kasadó ta tira e flecha	p	p	p	The hunter shoots the arrow

65	9	e muhè kust de man	e muhè kust de man	p	d	d	The woman kisses the man
66	9	de vrouw kust e hòmber	de vrouw kust e hòmber	d	d	p	The woman kisses the man
67	9	de vrouw kust de man	de vrouw kust de man	d	d	d	The woman kisses the man
68	9	e muhè kust e hombèr	e muhè kust e hombèr	p	d	p	The woman kisses the man
69	9	e muhè ta sunchi de man	e muhè ta sunchi de man	p	p	d	The woman kisses the man
70	9	de vrouw ta sunchi e hombèr	de vrouw ta sunchi e hombèr	d	p	p	The woman kisses the man
71	9	de vrouw ta sunchi de man	de vrouw ta sunchi de man	d	p	d	The woman kisses the man
72	9	e muhé ta sunchi e hòmber	e muhé ta sunchi e hòmber	p	p	p	The woman kisses the man
73	10	e enfermera meet de druk	e enfermera meet de druk	p	d	d	The nurses measures the pressure
74	10	de verpleegkundige meet e preshon	de verpleegkundige meet e preshon	d	d	p	The nurses measures the pressure
75	10	de verpleegkundige meet de druk	de verpleegkundige meet de druk	d	d	d	The nurses measures the pressure
76	10	e enfermera meet e preshon	e enfermera meet e preshon	p	d	p	The nurses measures the pressure
77	10	e enfermera ta midi de druk	e enfermera ta midi de druk	p	p	d	The nurses measures the pressure
78	10	de verpleegkundige ta midi e preshon	de verpleegkundige ta midi e preshon	d	p	p	The nurses measures the pressure
79	10	de verpleegkundige ta midi de druk	de verpleegkundige ta midi de druk	d	p	d	The nurses measures the pressure
80	10	e enfermera ta midi e preshon	e enfermera ta midi e preshon	p	p	p	The nurses measures the pressure
81	11	e drogadikto rookt de joint	e drogadikto rookt de joint	p	d	d	The addict smokes the joint
82	11	de verslaafde rookt e pitu	de verslaafde rookt e pitu	d	d	p	The addict smokes the joint
83	11	de verslaafde rookt de joint	de verslaafde rookt de joint	d	d	d	The addict smokes the joint
84	11	e drogadikto rookt e pitu	e drogadikto rookt e pitu	p	d	p	The addict smokes the joint
85	11	e drogadikto ta huma de joint	e drogadikto ta huma de joint	p	p	d	The addict smokes the joint
86	11	de verslaafde ta huma e pitu	de verslaafde ta huma e pitu	d	p	p	The addict smokes the joint
87	11	de verslaafde ta huma de joint	de verslaafde ta huma de joint	d	p	d	The addict smokes the joint
88	11	e drogadikto ta huma e pitu	e drogadikto ta huma e pitu	p	p	p	The addict smokes the joint
89	12	e kriá poetst de keuken	e kriá poetst de keuken	p	d	d	The cleaner cleans the kitchen
90	12	de hulp poetst e kushina	de hulp poetst e kushina	d	d	p	The cleaner cleans the kitchen
91	12	de hulp poetst de keuken	de hulp poetst de keuken	d	d	d	The cleaner cleans the kitchen
92	12	e kriá poetst e kushina	e kriá poetst e kushina	p	d	p	The cleaner cleans the kitchen
93	12	e kriá ta limpia de keuken	e kriá ta limpia de keuken	p	p	d	The cleaner cleans the kitchen
94	12	de hulp ta limpia e kushina	de hulp ta limpia e kushina	d	p	p	The cleaner cleans the kitchen
95	12	de hulp ta limpia de keuken	de hulp ta limpia de keuken	d	p	d	The cleaner cleans the kitchen
96	12	e kriá ta limpia e kushina	e kriá ta limpia e kushina	p	p	p	The cleaner cleans the kitchen
97	13	e mucha-hòmber eet de kip	e mucha-hòmber eet de kip	p	d	d	The boy eats the chicken
98	13	de jongen eet e galiña	de jongen eet e galiña	d	d	p	The boy eats the chicken

99	13	de jongen eet de kip	de jongen eet de kip	d	d	d	The boy eats the chicken
100	13	e mucha-hòmber eet e galiña	e mucha-hòmber eet e galiña	p	d	p	The boy eats the chicken
101	13	e mucha-hòmber ta kome de kip	e mucha-hòmber ta kome de kip	p	p	d	The boy eats the chicken
102	13	de jongen ta kome e galiña	de jongen ta kome e galiña	d	p	p	The boy eats the chicken
103	13	de jongen ta kome de kip	de jongen ta kome de kip	d	p	d	The boy eats the chicken
104	13	e mucha-hòmber ta kome e galiña	e mucha-hòmber ta kome e galiña	p	p	p	The boy eats the chicken
105	14	e kliente koopt de jurk	e kliente koopt de jurk	p	d	d	The customer buys the dress
106	14	de klant koopt e shimis	de klant koopt e shimis	d	d	p	The customer buys the dress
**107**	14	de klant koopt de jurk	de klant koopt de jurk	d	d	d	The customer buys the dress
108	14	e kliente koopt de shimis	e kliente koopt de shimis	p	d	p	The customer buys the dress
109	14	e kliente ta kumpra de jurk	e kliente ta kumpra de jurk	p	p	d	The customer buys the dress
110	14	de klant ta kumpra e shimis	de klant ta kumpra e shimis	d	p	p	The customer buys the dress
111	14	de klant ta kumpra de jurk	de klant ta kumpra de jurk	d	p	d	The customer buys the dress
112	14	e kliente ta kumpra e shimis	e kliente ta kumpra e shimis	p	p	p	The customer buys the dress
113	15	e señora geeft de melk	e señora geeft de melk	p	d	d	The lady gives the milk
114	15	de dame geeft e lechi	de dame geeft e lechi	d	d	p	The lady gives the milk
115	15	de dame geeft de melk	de dame geeft de melk	d	d	d	The lady gives the milk
116	15	e señora geeft e lechi	e señora geeft e lechi	p	d	p	The lady gives the milk
117	15	e señora ta duna de melk	e señora ta duna de melk	p	p	d	The lady gives the milk
118	15	de dame ta duna e lechi	de dame ta duna e lechi	d	p	p	The lady gives the milk
119	15	de dame ta duna de melk	de dame ta duna de melk	d	p	d	The lady gives the milk
120	15	e señora ta duna e lechi	e señora ta duna e lechi	p	p	p	The lady gives the milk
121	16	e ganado wint de taart	e ganado wint de taart	p	d	d	The winner wins the cake
122	16	de winnaar wint e bolo	de winnaar wint e bolo	d	d	p	The winner wins the cake
123	16	de winnaar wint de taart	de winnaar wint de taart	d	d	d	The winner wins the cake
124	16	e ganado wint e bolo	e ganado wint e bolo	p	d	p	The winner wins the cake
125	16	e ganado ta gana de taart	e ganado ta gana de taart	p	p	d	The winner wins the cake
126	16	de winnaar ta gana e bolo	de winnaar ta gana e bolo	d	p	p	The winner wins the cake
127	16	de winnaar ta gana de taart	de winnaar ta gana de taart	d	p	d	The winner wins the cake
128	16	e ganado ta gana e bolo	e ganado ta gana e bolo	p	p	p	The winner wins the cake
129	17	e amigo zoekt de sleutel	e amigo zoekt de sleutel	p	d	d	The friend searches for the key
130	17	de vriend zoekt e yabi	de vriend zoekt e yabi	d	d	p	The friend searches for the key
131	17	de vriend zoekt de sleutel	de vriend zoekt de sleutel	d	d	d	The friend searches for the key
132	17	e amigo zoekt e yabi	e amigo zoekt e yabi	p	d	p	The friend searches for the key

133	17	e amigo ta buska de sleutel	e amigo ta buska de sleutel	p	p	d	The friend searches for the key
134	17	de vriend ta buska e yabi	de vriend ta buska e yabi	d	p	p	The friend searches for the key
135	17	de vriend ta buska de sleutel	de vriend ta buska de sleutel	d	p	d	The friend searches for the key
136	17	e amigo ta buska e yabi	e amigo ta buska e yabi	p	p	p	The friend searches for the key
137	18	e kantante zingt de (song)tekst	e kantante zingt de (song)tekst	p	d	d	The singer sings the lyrics
138	18	de zanger zingt e letra	de zanger zingt e letra	d	d	p	The singer sings the lyrics
139	18	de zanger zingt de (song)tekst	de zanger zingt de (song)tekst	d	d	d	The singer sings the lyrics
140	18	e kantante zingt e letra	e kantante zingt e letra	p	d	p	The singer sings the lyrics
141	18	e kantante ta kanta de (song)tekst	e kantante ta kanta de (song)tekst	p	p	d	The singer sings the lyrics
142	18	de zanger ta kanta e letra	de zanger ta kanta e letra	d	p	p	The singer sings the lyrics
143	18	de zanger ta kanta de (song)tekst	de zanger ta kanta de (song)tekst	d	p	d	The singer sings the lyrics
144	18	e kantante ta kanta e letra	e kantante ta kanta e letra	p	p	p	The singer sings the lyrics
145	19	e para pakt de slang	e para pakt de slang	p	d	d	The bird catches the snake
146	19	de vogel pakt e kolebra	de vogel pakt e kolebra	d	d	p	The bird catches the snake
147	19	de vogel pakt de slang	de vogel pakt de slang	d	d	d	The bird catches the snake
148	19	e para pakt e kolebra	e para pakt e kolebra	p	d	p	The bird catches the snake
149	19	e para ta gara de slang	e para ta gara de slang	p	p	d	The bird catches the snake
150	19	de vogel ta gara e kolebra	de vogel ta gara e kolebra	d	p	p	The bird catches the snake
151	19	de vogel ta gara de slang	de vogel ta gara de slang	d	p	d	The bird catches the snake
152	19	e para ta gara e kolebra	e para ta gara e kolebra	p	p	p	The bird catches the snake
153	20	e ekstranhero leert de taal	e ekstranhero leert de taal	p	d	d	The foreigner learns the language
154	20	de buitenlander leert e idioma	de buitenlander leert e idioma	d	d	p	The foreigner learns the language
155	20	de buitenlander leert de taal	de buitenlander leert de taal	d	d	d	The foreigner learns the language
156	20	e ekstranhero leert e idioma	e ekstranhero leert e idioma	p	d	p	The foreigner learns the language
157	20	e ekstranhero ta siña de taal	e ekstranhero ta siña de taal	p	p	d	The foreigner learns the language
158	20	de buitenlander ta siña e idioma	de buitenlander ta siña e idioma	d	p	p	The foreigner learns the language
159	20	de buitenlander ta siña de taal	de buitenlander ta siña de taal	d	p	d	The foreigner learns the language
160	20	e ekstranhero ta siña e idioma	e ekstranhero ta siña e idioma	p	p	p	The foreigner learns the language
A	F	De kinderen gaan naar de feesten	De jongens gaan naar de feesten	NP.pl	V	PP.pl	The kids/boys go to the parties
B	F	Het meisje liep naar de deur	Het meisje liep naar de kast	NP.sg	V	NP.pl	The girl walked to the door/closet
C	F	De mannen drinken wijn	De mannen drinken bier	NP.pl	V	NP.sg	The men drink wine/beer
D	F	E yunan ta bai e fiestanan	E yunan ta bai fiesta	NP.sg	V	NP	The kids go to the party/go partying
E	F	E mucha muhé a kana bai porta	E mucha hombèr a kana bai porta	NP.sg	V	PP.sg	The girl/boy walked to the door
F	F	E musiko tabata toka violin	E musiko tabata toka tròmpèt	NP.sg	V	NP.sg	The musician plays violin/trumpet

Stimuli

G	F	Nos a paga het avondeten	Wij a paga het avondeten	NP.pl	V	NP.sg	We are eating dinner
H	F	E piskadónan a fangu hopi vis	E piskadónan a fangu hopi piská	NP.sg	V	NP.sg	The fisher catches a lot of fish
I	F	De man praat hopi idioma	E hòmber praat hopi idioma	NP.sg	V	NP.pl	The man speaks a lot of languages
J	F	E hugadónan hebben hopi ruzie	E hugadónan tin hopi ruzie	NP.sg	V	NP.pl	The players are fighting
P1	P	E laman di Hulanda ta hopi fríu.	E laman di Hulanda ta hopi koud.	NP.sg	V	Adv	The Dutch sea is very cold
P2	P	Mi bisinja heeft un barba maron.	Mi bisinja heeft un barba maron.	NP	V	NP.sg	My neighbour has a brown beard
P3	P	Mi amigu drinkt awa lamunchi.	Mi amigu drinkt cola.	NP.sg	V	Adv/NP	My friend drinks a lot/cola
