# Supplementary material for: Structural and Extralinguistic Aspects of Code-Switching: Evidence From Papiamentu-Dutch Auditory Sentence Matching
Source: Front Psychol. 2020 Dec 22;11:592266. doi: 10.3389/fpsyg.2020.592266 (PMC7783355; doi:10.3389/fpsyg.2020.592266)
Supplement: Supplementary file 3 [file Table_3.PDF]

Mixed-effects regression model outputs
Switch direction

```
## Linear mixed model fit by maximum likelihood . t-tests use Satterthwaite's
##   method [lmerModLmerTest]
## Formula: logRT ~ direction + (1 | participant) + (1 | ID)
##    Data: df
##
##      AIC      BIC   logLik deviance df.resid
##    818.7    839.3   -404.4    808.7      447
##
## Scaled residuals:
##     Min      1Q  Median      3Q     Max
## -3.4460 -0.5856 -0.1269  0.5482  2.9513
##
## Random effects:
##  Groups      Name        Variance  Std.Dev.
##  ID          (Intercept) 2.436e-10 1.561e-05
##  participant (Intercept) 1.315e-01 3.627e-01
##  Residual                2.930e-01 5.413e-01
## Number of obs: 452, groups:  ID, 85; participant, 50
##
## Fixed effects:
##              Estimate Std. Error        df t value Pr(>|t|)
## (Intercept)  -0.65453    0.06271  70.48327 -10.438 6.01e-16 ***
## direction2   -0.10401    0.05122 403.96484  -2.031   0.0429 *
## ---
## Signif. codes:  0 '***' 0.001 '**' 0.01 '*' 0.05 '.' 0.1 ' ' 1
##
## Correlation of Fixed Effects:
##            (Intr)
## direction2 -0.406
## convergence code: 0
## boundary (singular) fit: see ?isSingular
```

```
## Data: df
## Models:
## m0: logRT ~ 1 + (1 | participant) + (1 | ID)
## m2: logRT ~ direction + (1 | participant) + (1 | ID)
##    npar    AIC    BIC  logLik deviance Chisq Df Pr(>Chisq)
## m0    4 820.80 837.25 -406.40   812.80
## m2    5 818.73 839.30 -404.36   808.73  4.07  1    0.04365 *
## ---
## Signif. codes:  0 '***' 0.001 '**' 0.01 '*' 0.05 '.' 0.1 ' ' 1
```

Switch location

```
## Linear mixed model fit by maximum likelihood . t-tests use Satterthwaite's
##   method [lmerModLmerTest]
## Formula: logRT ~ location + (1 | participant) + (1 | ID)
##    Data: df
##
##      AIC      BIC   logLik deviance df.resid
##    822.8    843.4   -406.4    812.8      447
##
## Scaled residuals:
##     Min      1Q  Median      3Q     Max
## -3.5120 -0.5592 -0.1099  0.5181  3.0102
##
## Random effects:
##  Groups      Name        Variance Std.Dev.
##  ID          (Intercept) 0.001663 0.04079
##  participant (Intercept) 0.129900 0.36042
##  Residual                0.294601 0.54277
## Number of obs: 452, groups:  ID, 85; participant, 50
##
## Fixed effects:
##              Estimate Std. Error        df t value Pr(>|t|)
## (Intercept) -0.704843   0.062439 64.720620 -11.289   <2e-16 ***
## location2   -0.002805   0.052358 70.679374  -0.054    0.957
## ---
## Signif. codes:  0 '***' 0.001 '**' 0.01 '*' 0.05 '.' 0.1 ' ' 1
##
## Correlation of Fixed Effects:
##           (Intr)
## location2 -0.400
```

```
## Data: df
## Models:
## m0: logRT ~ 1 + (1 | participant) + (1 | ID)
## m2: logRT ~ location + (1 | participant) + (1 | ID)
##    npar    AIC    BIC logLik deviance  Chisq Df Pr(>Chisq)
## m0    4 820.80 837.25 -406.4   812.80
## m2    5 822.79 843.36 -406.4   812.79 0.0029  1     0.9573
```

Interaction between switch location and direction

```
## Linear mixed model fit by maximum likelihood . t-tests use Satterthwaite's
##   method [lmerModLmerTest]
## Formula: logRT ~ location:direction + (1 | participant) + (1 | ID)
##    Data: df
##
##      AIC      BIC   logLik deviance df.resid
##    821.6    850.3   -403.8    807.6      445
##
## Scaled residuals:
##     Min      1Q  Median      3Q     Max
## -3.5036 -0.5839 -0.1073  0.5080  2.9131
##
## Random effects:
##  Groups      Name        Variance  Std.Dev.
##  ID          (Intercept) 8.104e-05 0.009002
##  participant (Intercept) 1.312e-01 0.362253
##  Residual                2.921e-01 0.540490
## Number of obs: 452, groups:  ID, 85; participant, 50
##
## Fixed effects:
##                      Estimate Std. Error       df t value Pr(>|t|)
## (Intercept)          -0.79080    0.07385 99.55468 -10.708   <2e-16 ***
## location1:direction1  0.11156    0.07319 75.90944   1.524   0.1316
## location2:direction1  0.16262    0.07449 82.85173   2.183   0.0319 *
## location1:direction2  0.06070    0.07319 75.90757   0.829   0.4095
## ---
## Signif. codes:  0 '***' 0.001 '**' 0.01 '*' 0.05 '.' 0.1 ' ' 1
##
## Correlation of Fixed Effects:
##             (Intr) lct1:1 lct2:1
## lctn1:drct1 -0.523
## lctn2:drct1 -0.516  0.517
## lctn1:drct2 -0.527  0.529  0.526
## fit warnings:
## fixed-effect model matrix is rank deficient so dropping 1 column / coefficient
```

```
## Data: df
## Models:
## m0: logRT ~ 1 + (1 | participant) + (1 | ID)
## m2: logRT ~ location:direction + (1 | participant) + (1 | ID)
##    npar    AIC    BIC  logLik deviance  Chisq Df Pr(>Chisq)
## m0    4 820.80 837.25 -406.40   812.80
## m2    7 821.55 850.35 -403.78   807.55 5.2442  3     0.1548
```

Habits as random effect, direction as fixed effect

```
## Linear mixed model fit by maximum likelihood . t-tests use Satterthwaite's
##   method [lmerModLmerTest]
## Formula: logRT ~ direction + (1 | participant) + (1 | ID) + (1 | habits)
##    Data: df_habits
##
##      AIC      BIC   logLik deviance df.resid
##    716.3    740.4   -352.2    704.3      403
##
## Scaled residuals:
##     Min      1Q  Median      3Q     Max
## -3.4495 -0.5694 -0.1317  0.5633  2.9458
##
## Random effects:
##  Groups      Name        Variance Std.Dev.
##  ID          (Intercept) 0.01041  0.1020
##  participant (Intercept) 0.11130  0.3336
##  habits      (Intercept) 0.00000  0.0000
##  Residual                0.26706  0.5168
## Number of obs: 409, groups:  ID, 85; participant, 45; habits, 5
##
## Fixed effects:
##             Estimate Std. Error       df t value Pr(>|t|)
## (Intercept) -0.65084    0.06359 65.30001 -10.234 3.34e-15 ***
## direction2  -0.09395    0.05605 76.37880  -1.676   0.0978 .
## ---
## Signif. codes:  0 '***' 0.001 '**' 0.01 '*' 0.05 '.' 0.1 ' ' 1
##
## Correlation of Fixed Effects:
##            (Intr)
## direction2 -0.439
## convergence code: 0
## boundary (singular) fit: see ?isSingular
```

Habits as random effect, location as fixed effect

```
## Linear mixed model fit by maximum likelihood . t-tests use Satterthwaite's
##   method [lmerModLmerTest]
## Formula: logRT ~ location + (1 | participant) + (1 | ID) + (1 | habits)
##    Data: df_habits
##
##      AIC      BIC   logLik deviance df.resid
##    719.1    743.2   -353.6    707.1      403
##
## Scaled residuals:
##     Min      1Q  Median      3Q     Max
## -3.4987 -0.5460 -0.1193  0.5278  3.0008
##
## Random effects:
##  Groups      Name        Variance Std.Dev.
##  ID          (Intercept) 0.01196  0.1094
##  participant (Intercept) 0.11061  0.3326
##  habits      (Intercept) 0.00000  0.0000
##  Residual                0.26795  0.5176
## Number of obs: 409, groups:  ID, 85; participant, 45; habits, 5
##
## Fixed effects:
##              Estimate Std. Error        df t value Pr(>|t|)
## (Intercept) -0.696870   0.063464 64.203003 -10.981 2.23e-16 ***
## location2   -0.001608   0.057236 69.536665  -0.028    0.978
## ---
## Signif. codes:  0 '***' 0.001 '**' 0.01 '*' 0.05 '.' 0.1 ' ' 1
##
## Correlation of Fixed Effects:
##           (Intr)
## location2 -0.434
## convergence code: 0
## boundary (singular) fit: see ?isSingular
```

Attitudes as random effect, direction as fixed effect

```
## Linear mixed model fit by maximum likelihood . t-tests use Satterthwaite's
##   method [lmerModLmerTest]
## Formula: logRT ~ direction + (1 | participant) + (1 | ID) + (1 | attitudes)
##    Data: df_att
##
##      AIC      BIC   logLik deviance df.resid
##    716.3    740.4   -352.2    704.3      403
##
## Scaled residuals:
##     Min      1Q  Median      3Q     Max
## -3.4495 -0.5694 -0.1317  0.5633  2.9458
##
## Random effects:
##  Groups      Name        Variance Std.Dev.
##  ID          (Intercept) 0.01041  0.1020
##  participant (Intercept) 0.11130  0.3336
##  attitudes   (Intercept) 0.00000  0.0000
##  Residual                0.26706  0.5168
## Number of obs: 409, groups:  ID, 85; participant, 45; attitudes, 5
##
## Fixed effects:
##             Estimate Std. Error       df t value Pr(>|t|)
## (Intercept) -0.65084    0.06359 65.30103 -10.234 3.33e-15 ***
## direction2  -0.09395    0.05605 76.37888  -1.676   0.0978 .
## ---
## Signif. codes:  0 '***' 0.001 '**' 0.01 '*' 0.05 '.' 0.1 ' ' 1
##
## Correlation of Fixed Effects:
##            (Intr)
## direction2 -0.439
## convergence code: 0
## boundary (singular) fit: see ?isSingular
```

Attitudes as random effect, location as fixed effect

```
## Linear mixed model fit by maximum likelihood . t-tests use Satterthwaite's
##   method [lmerModLmerTest]
## Formula: logRT ~ location + (1 | participant) + (1 | ID) + (1 | attitudes)
##    Data: df_att
##
##      AIC      BIC   logLik deviance df.resid
##    719.1    743.2   -353.6    707.1      403
##
## Scaled residuals:
##     Min      1Q  Median      3Q     Max
## -3.4987 -0.5460 -0.1193  0.5278  3.0008
##
## Random effects:
##  Groups      Name        Variance Std.Dev.
##  ID          (Intercept) 0.01196  0.1094
##  participant (Intercept) 0.11061  0.3326
##  attitudes   (Intercept) 0.00000  0.0000
##  Residual                0.26795  0.5176
## Number of obs: 409, groups:  ID, 85; participant, 45; attitudes, 5
##
## Fixed effects:
##              Estimate Std. Error        df t value Pr(>|t|)
## (Intercept) -0.696870   0.063464 64.203105 -10.981 2.23e-16 ***
## location2   -0.001608   0.057236 69.536746  -0.028    0.978
## ---
## Signif. codes:  0 '***' 0.001 '**' 0.01 '*' 0.05 '.' 0.1 ' ' 1
##
## Correlation of Fixed Effects:
##           (Intr)
## location2 -0.434
## convergence code: 0
## boundary (singular) fit: see ?isSingular
```

Papiamento ability as random effect, direction as fixed effect

```
## Linear mixed model fit by maximum likelihood . t-tests use Satterthwaite's
##   method [lmerModLmerTest]
## Formula: logRT ~ direction + (1 | participant) + (1 | ID) + (1 | `pap ability`)
##    Data: df_pap
##
##      AIC      BIC   logLik deviance df.resid
##    746.6    770.8   -367.3    734.6      410
##
## Scaled residuals:
##     Min      1Q  Median      3Q     Max
## -3.4597 -0.5892 -0.1313  0.5624  2.9788
##
## Random effects:
##  Groups      Name        Variance  Std.Dev.
##  ID          (Intercept) 0.000e+00 0.000e+00
##  participant (Intercept) 1.155e-01 3.398e-01
##  pap ability (Intercept) 3.000e-11 5.478e-06
##  Residual                2.892e-01 5.378e-01
## Number of obs: 416, groups:  ID, 85; participant, 46; pap ability, 4
##
## Fixed effects:
##              Estimate Std. Error        df t value Pr(>|t|)
## (Intercept)  -0.66300    0.06248  66.97197 -10.611 5.53e-16 ***
## direction2   -0.09367    0.05302 372.06423  -1.767   0.0781 .
## ---
## Signif. codes:  0 '***' 0.001 '**' 0.01 '*' 0.05 '.' 0.1 ' ' 1
##
## Correlation of Fixed Effects:
##            (Intr)
## direction2 -0.422
## convergence code: 0
## boundary (singular) fit: see ?isSingular
```

Papiamento ability as random effect, location as fixed effect

```
## Linear mixed model fit by maximum likelihood . t-tests use Satterthwaite's
##   method [lmerModLmerTest]
## Formula: logRT ~ location + (1 | participant) + (1 | ID) + (1 | `pap ability`)
##    Data: df_pap
##
##      AIC      BIC   logLik deviance df.resid
##    749.7    773.9   -368.9    737.7      410
##
## Scaled residuals:
##     Min      1Q  Median      3Q     Max
## -3.5313 -0.5827 -0.1131  0.5498  3.0544
##
## Random effects:
##  Groups      Name        Variance  Std.Dev.
##  ID          (Intercept) 0.000e+00 0.000e+00
##  participant (Intercept) 1.146e-01 3.386e-01
##  pap ability (Intercept) 5.007e-11 7.076e-06
##  Residual                2.917e-01 5.401e-01
## Number of obs: 416, groups:  ID, 85; participant, 46; pap ability, 4
##
## Fixed effects:
##               Estimate Std. Error         df t value Pr(>|t|)
## (Intercept)  -0.708223   0.061982  65.379425 -11.426   <2e-16 ***
## location2    -0.002716   0.053489 373.375003  -0.051     0.96
## ---
## Signif. codes:  0 '***' 0.001 '**' 0.01 '*' 0.05 '.' 0.1 ' ' 1
##
## Correlation of Fixed Effects:
##           (Intr)
## location2 -0.409
## convergence code: 0
## boundary (singular) fit: see ?isSingular
```

Dutch ability as random effect, direction as fixed effect

```
## Linear mixed model fit by maximum likelihood . t-tests use Satterthwaite's
##   method [lmerModLmerTest]
## Formula:
## logRT ~ direction + (1 | participant) + (1 | ID) + (1 | `dutch ability`)
##    Data: df_dutch
##
##      AIC      BIC   logLik deviance df.resid
##    716.3    740.4   -352.2    704.3      403
##
## Scaled residuals:
##     Min      1Q  Median      3Q     Max
## -3.4495 -0.5694 -0.1317  0.5633  2.9458
##
## Random effects:
##  Groups        Name        Variance Std.Dev.
##  ID            (Intercept) 0.01041  0.1020
##  participant   (Intercept) 0.11130  0.3336
##  dutch ability (Intercept) 0.00000  0.0000
##  Residual                  0.26706  0.5168
## Number of obs: 409, groups:  ID, 85; participant, 45; dutch ability, 4
##
## Fixed effects:
##             Estimate Std. Error       df t value Pr(>|t|)
## (Intercept) -0.65084    0.06359 65.30073 -10.234 3.34e-15 ***
## direction2  -0.09395    0.05605 76.37885  -1.676   0.0978 .
## ---
## Signif. codes:  0 '***' 0.001 '**' 0.01 '*' 0.05 '.' 0.1 ' ' 1
##
## Correlation of Fixed Effects:
##            (Intr)
## direction2 -0.439
## convergence code: 0
## boundary (singular) fit: see ?isSingular
```

Dutch ability as random effect, location as fixed effect

```
## Linear mixed model fit by maximum likelihood . t-tests use Satterthwaite's
##   method [lmerModLmerTest]
## Formula:
## logRT ~ location + (1 | participant) + (1 | ID) + (1 | `dutch ability`)
##    Data: df_dutch
##
##      AIC      BIC   logLik deviance df.resid
##    719.1    743.2   -353.6    707.1      403
##
## Scaled residuals:
##     Min      1Q  Median      3Q     Max
## -3.4987 -0.5460 -0.1193  0.5278  3.0008
##
## Random effects:
##  Groups        Name        Variance Std.Dev.
##  ID            (Intercept) 0.01196  0.1094
##  participant   (Intercept) 0.11061  0.3326
##  dutch ability (Intercept) 0.00000  0.0000
##  Residual                  0.26795  0.5176
## Number of obs: 409, groups:  ID, 85; participant, 45; dutch ability, 4
##
## Fixed effects:
##              Estimate Std. Error        df t value Pr(>|t|)
## (Intercept) -0.696870   0.063464 64.203447 -10.981 2.23e-16 ***
## location2   -0.001608   0.057236 69.536735  -0.028    0.978
## ---
## Signif. codes:  0 '***' 0.001 '**' 0.01 '*' 0.05 '.' 0.1 ' ' 1
##
## Correlation of Fixed Effects:
##           (Intr)
## location2 -0.434
## convergence code: 0
## boundary (singular) fit: see ?isSingular
```
